# Low-Dose Gallic Acid Administration Does Not Improve Diet-Induced Metabolic Disorders and Atherosclerosis in Apoe Knockout Mice

**DOI:** 10.1155/2022/7909971

**Published:** 2022-05-23

**Authors:** Jie Bai, Qiu-Yue Lin, Xiangbo An, Shuang Liu, Yao Wang, Yunpeng Xie, Jiawei Liao

**Affiliations:** ^1^Institute of Cardiovascular Diseases, First Affiliated Hospital of Dalian Medical University, Dalian 116011, China; ^2^Department of Interventional Therapy, First Affiliated Hospital of Dalian Medical University, Dalian 116011, China; ^3^College of Basic Medical Sciences, Dalian Medical University, Dalian 116044, China

## Abstract

Diets rich in polyphenols are known to be beneficial for cardiovascular health. Gallic acid (GA) is a plant-derived triphenolic chemical with multiple cardio-protective properties, such as antiobesity, anti-inflammation, and antioxidation. However, whether GA could protect against atherosclerotic cardiovascular diseases is still not defined. Here, we investigated the effects of low-dose GA administration on diet-induced metabolic disorders and atherosclerosis in the atherosclerosis-prone apolipoprotein E (Apoe) knockout mice fed on a high-fat Western-type diet (WTD) for 8 weeks. Our data showed that GA administration by oral gavage at a daily dosage of 20 mg/kg body weight did not significantly ameliorate WTD-induced hyperlipidemia, hepatosteatosis, adipogenesis, or insulin resistance; furthermore, GA administration did not significantly ameliorate WTD-induced atherosclerosis. In conclusion, our data demonstrate that low-dose GA administration does not elicit significant health effect on diet-induced metabolic disorders or atherosclerosis in the Apoe knockout mice. Whether GA could be beneficial for atherosclerotic cardiovascular diseases therefore needs further exploration.

## 1. Introduction

Cardiovascular diseases, especially atherosclerotic cardiovascular diseases (ASCVDs), are the leading cause of global mortality and morbidity [1]. Metabolic disorders, such as hyperlipidemia, insulin resistance, and obesity, are well-known risk factors of ASCVDs. In addition to these traditional determinants, lifestyle factors are increasingly recognized of their impacts on metabolic homeostasis and disease progression through several intermediary pathways, such as inflammation and oxidative stress [2].

Diet management is an important aspect of lifestyle modification. Mounting epidemiological and experimental evidence has shown that diets rich in plant-derived polyphenols, such as isoflavone and resveratrol, are beneficial for cardiometabolic health, due to their multiple pharmacological properties, including hypolipidemic, antiobese, anti-inflammatory, and antioxidative activities [3, 4]. Exploration of the cardio-protective effects of polyphenolic compounds therefore stands as a hot and long-lasting research interest over the past few decades.

Gallic acid (GA) or 3,4,5-trihydroxybenzoic acid, with the molecular formula C_7_H_6_O_5_ (MW 170.12 g/mol), is a low molecular weight triphenolic molecule that found abundant in nuts, tea, and various fruits (such as grapes, strawberries, and bananas) [5]. Well known for its antioxidative, anti-inflammatory, and radioprotective properties, GA has been used as food additives and cosmetics [5]. In addition to its broad-spectrum industrial applications, GA also exhibits a promising pharmaceutical potent against cardiovascular diseases. For example, in a rat model of streptozotocin-induced diabetes, GA effectively prevents cardiac remodeling and dysfunction by improving glucose/lipid metabolism and reducing oxidative stress [6]; furthermore, in mouse models of cardiac remodeling and failure induced either by isoproterenol or pressure overload, GA has been demonstrated to attenuate cardiac hypertrophy and fibrosis through multiple signaling pathways [7–9]. However, whether GA could produce beneficial effects on maintaining cardiometabolic homeostasis and preventing ASCVD are still not defined. In this study, we explored this issue in the apolipoprotein E (Apoe) knockout (KO) mice fed on a high-fat Western-type diet (WTD).

## 2. Materials and Methods

### 2.1. Animals, Diet, and Experimental Design

Male Apoe KO mice (C57BL/6 background) aged 6-7 weeks old were purchased from Beijing Vital River Laboratory. Mice were housed under specific-pathogen-free conditions on a 12-hour light/12-hour dark cycle and fed with laboratory rodent chow and sterilized water *ad libitum*. After 1-week adaptation, mice were randomly divided into GA group and control group (*n* = 8 per group). Mice in the GA group received oral administrations of GA (MedChemExpress, USA) at a daily dosage of 20 mg/kg body weight, while those in the control group received the same amount of saline. Both groups were fed with a high-fat Western-type diet (WTD) containing 0.15% cholesterol and 20% fat for eight weeks to induce metabolic disorders and atherogenesis. All experimental procedures were performed in accordance with the Guide for the Care and Use of Laboratory Animals and approved by the Animal Care and Use Committee of Dalian Medical University.

### 2.2. Plasma Lipid and Lipoprotein Profile Analysis

Blood samples were collected by retro-orbital bleeding after mice were fasted for 4 hours. Plasma total cholesterol (TC), triglycerides (TG), high-density lipoprotein cholesterol (HDL-C), and low-density lipoprotein cholesterol (LDL-C) levels were measured with commercial kits (BioSino, China), according to the manufacturer's guidance. Lipoprotein profile analysis was performed as we previously described [10]. Briefly, pooled plasma samples were applied to Tricorn high-performance Superose S-6 10/300 GL columns and fractioned by a fast protein liquid chromatography (FPLC) system (Amersham Biosciences, UK). Cholesterol concentration in each eluted fraction was measured with commercial kit (BioSino, China).

### 2.3. Plasma Glucose and Glucose Tolerance Analysis

Plasma glucose levels of fasted blood samples were measured with commercial kit (BioSino, China), according to the manufacturer's guidance. For glucose tolerance test, mice were fasted for 4 hours and given glucose (Abbott, USA) at a dosage of 2 g/kg body weight via intraperitoneal injection [11]. Blood samples were collected before (time 0) and at 15, 30, 60, and 120 minutes after glucose injection. Plasma glucose levels in different time points were measured as described above.

### 2.4. Hepatic Lipid Analysis

After 8 weeks on the WTD feeding, mice were sacrificed by lethal-dose anesthesia and flushed with PBS through the left ventricle. The livers were removed and weighted. For histological analysis, the livers were fixed in 4% paraformaldehyde (Life-iLab, Shanghai, China), embedded in OCT (Sakura Finetek, USA) and cross-sectioned at 7 *μ*m thickness. Hepatic lipids were visualized by Oil-red O (Sigma, USA) staining. Quantification of hepatic lipid contents was performed as previously described [10]. Briefly, liver samples (approximately 100 mg) were weighed and homogenized in 1 ml PBS. Lipids were extracted using Folch's reagent and dissolved in 1 ml 3% Triton X-100. TC and TG contents in the solutions were measured with commercial kits (BioSino, China) described above and then normalized to liver weight.

### 2.5. Adipose Tissue Analysis

After mice were sacrificed and flushed, subcutaneous white adipose tissue (WAT), epididymal WAT, mesenteric WAT, and retroperitoneal WAT were removed and weighted. For histological analysis, epididymal WAT was fixed in 4% paraformaldehyde (Life-iLab, Shanghai, China), embedded in paraffin, and cross-sectioned at 7 *μ*m thickness. Adipocyte morphology was visualized by hematoxylin and eosin (H&E) staining.

### 2.6. Quantitative Real-Time PCR Analysis

Total RNA was extracted and reverse-transcripted to complementary DNA as we previously described [12]. Quantitative real-time PCR was performed using SYBR Green PCR reagents (MedChemExpress, USA). Samples were quantitated by the comparative CT method for relative quantitation and normalized to Gapdh. The primer sequences used in the experiments are described in Table 1.

### 2.7. Atherosclerosis Analysis

The hearts were fixed in 4% paraformaldehyde (Life-iLab, Shanghai, China), embedded in OCT (Sakura Finetek, USA), and cross-sectioned at 7 *μ*m thickness as we previously described [12]. Briefly, cytosections were collected from the point where all the three aortic valve cusps were clearly visible, and 5-6 sections, each separated by 70 *μ*m of the tissue, were included into quantification. Atherosclerosis burden in the aortic root was visualized by Oil-red O (Sigma, USA) staining. Infiltration of inflammatory macrophages in the atherosclerotic plaques was visualized by immunohistochemical staining with anti-CD68 antibody (MCA1957, diluted at 1 : 200; Bio-Rad, USA). Plaque oxidative stress was visualized by dihydroethidium (DHE, 1 *μ*M) staining. Quantifications were performed with the ImageJ software.

### 2.8. Statistical Analysis

Statistical analysis was performed with the Prism software and presented as Mean ± SEM. Significance was evaluated by Student's *t*-test or one-way ANOVA. A *P* value < 0.05 was considered significant.

## 3. Results

### 3.1. Low-Dose GA Administration Produces No Significant Benefit against Diet-Induced Hyperlipidemia

**Figure 1:**
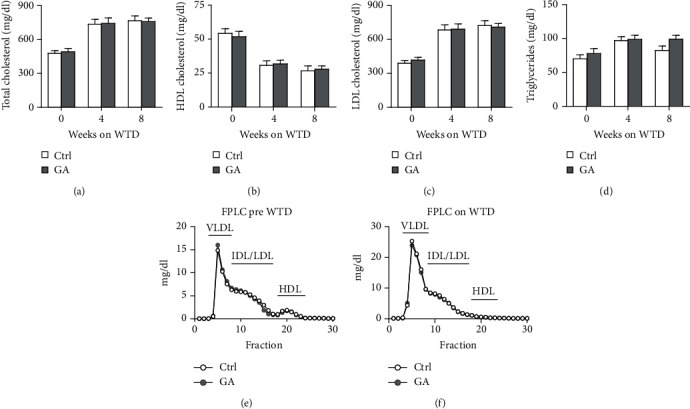
Effect of low-dose GA administration on diet-induced hyperlipidemia in the Apoe KO mice. (a–d) Plasma total cholesterol (a), high-density lipoprotein cholesterol (b), low-density lipoprotein cholesterol (c), and triglycerides (d) levels during WTD feeding, *n* = 8 per group. (e, f) Plasma lipoprotein profiles fractioned by a fast protein liquid chromatography (FPLC) before and after 8 weeks on WTD feeding. Samples were pooled from 5 mice of the same group.

To explore whether low-dose GA administration had any beneficial effect on diet-induced hyperlipidemia, fasting plasma of the Apoe KO mice was collected, and the lipid levels were compared between GA-administrated group and saline-administrated controls. Our data showed that low-dose GA administration did not exert any significant effects on plasma total cholesterol (TC), high-density lipoprotein cholesterol (HDL-C), low-density lipoprotein cholesterol (LDL-C), or triglyceride (TG) levels during high-fat WTD feeding (Figures 1(a)–1(d)); similarly, low-dose GA administration did not affect diet-induced change of plasma lipoprotein profiles, as shown by FPLC analysis (Figures 1(e) and 1(f)).

### 3.2. Low-Dose GA Administration Produces No Significant Benefit against Diet-Induced Hepatosteatosis

**Figure 2:**
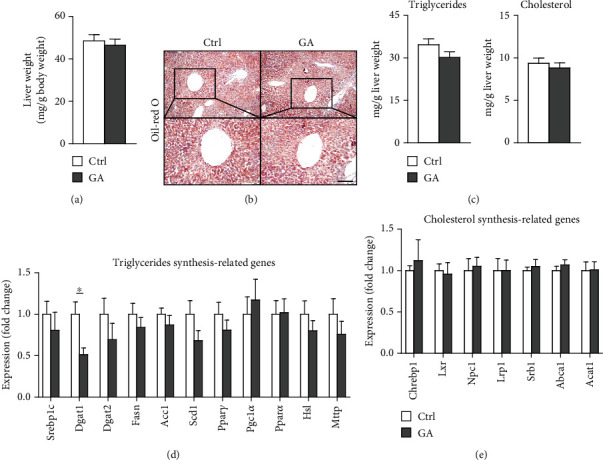
Effect of low-dose GA administration on diet-induced hepatosteatosis in the Apoe KO mice. (a) Liver weight. (b) Representative Oil-red O staining images of liver section. The bar represents 100 *μ*m. (c) Quantitation of hepatic triglyceride and total cholesterol content. (d, e) Quantitative real-time PCR analysis of triglycerides synthesis-related (d) and cholesterol synthesis-related (e) gene expression in the liver. *n* = 6-8 per group. ^∗^*p* < 0.05.

High-fat WTD feeding induces not only hyperlipidemia but also hepatic fat deposition that eventually leads to hepatosteatosis. Therefore, we explored the effects of GA administration on hepatic lipid metabolism. Our data showed that low-dose GA administration did not change liver weight gain after WTD feeding for 8 weeks (Figure 2(a)). Oil-red O staining showed that low-dose GA administration did not reduce hepatic lipid deposition (Figure 2(b)), which were further confirmed by lipid extraction (Figure 2(c)). Using quantitative real-time PCR analysis, we demonstrated that low-dose GA administration did not change the expression of genes related to hepatic triglyceride synthesis, such as Dgat1, Fasn, and Scd1 (Figure 2(d)), as well as those related to hepatic cholesterol synthesis, such as Lrp1, Srb1, and Abca1 (Figure 2(e)).

### 3.3. Low-Dose GA Administration Produces No Significant Benefit against Diet-Induced Adipogenesis

**Figure 3:**
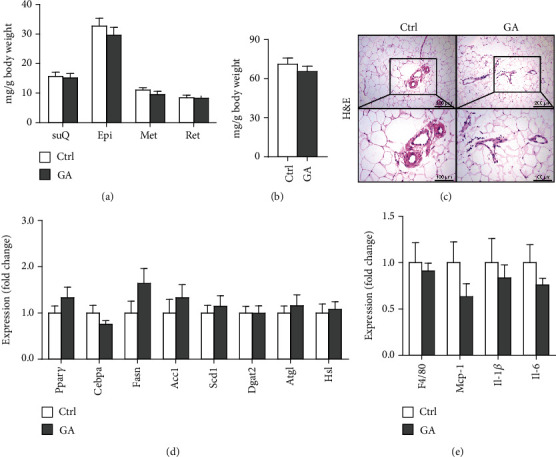
Effect of low-dose GA administration on diet-induced adipogenesis in the Apoe KO mice. (a, b) Weight of WATs, measured by counting weight of subcutaneous (suQ), epididymal (Epi), mesenteric (Met), and retroperitoneal (Ret) WAT separately (a) or together (b). (c) Representative H&E staining images of Epi WAT section. (d) Quantitative real-time PCR analysis of adipogenesis-related gene expression in Epi WAT. (e) Quantitative real-time PCR analysis of inflammation-related gene expression in Epi WAT. *n* = 6-8 per group.

White adipose tissues (WATs) would expand in sizes responding to high-fat WTD feeding, a process known as adipogenesis. This diet-induced adipogenesis process is often associated with adipose inflammation, which could further contribute to diet-induced insulin resistance and obesity. After feeding Apoe KO mice with the high-fat WTD for 8 weeks, we found that low-dose GA administration did not attenuate diet-induced expansion of WATs, measured by counting subcutaneous WAT, epididymal WAT, mesenteric WAT, and retroperitoneal WAT, the major four white adipose depots separately (Figure 3(a)) or together (Figure 3(b)). H&E staining further showed that low-dose GA administration did not cause any significant change of adipocyte morphology (Figure 3(c)). Real-time PCR analysis showed that low-dose GA administration had no significant impact on the expression of adipogenesis-related genes, such as Cebpa, Fasn, Scd1, and Atgl (Figure 3(d)), as well as inflammation-associated genes, such as Mcp-1, Il-1*β*, and Il-6 (Figure 3(e)).

### 3.4. Low-Dose GA Administration Produces No Significant Benefit against Diet-Induced Insulin Resistance

**Figure 4:**
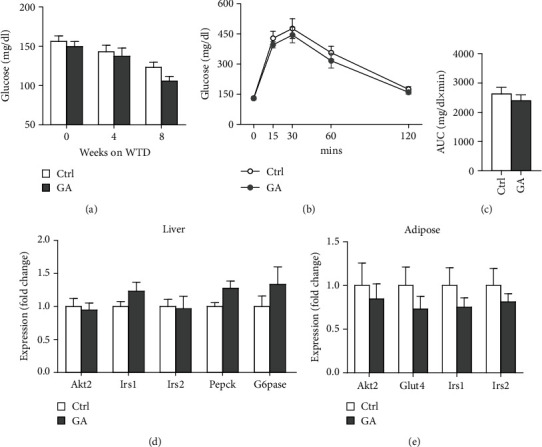
Effect of low-dose GA administration on diet-induced insulin resistance in the Apoe KO mice. (a) Plasma glucose levels during WTD feeding. (b, c) Glucose tolerance test after 6 weeks on WTD feeding (b) and quantitation of area under curve (c). (d, e) Quantitative real-time PCR analysis of insulin resistance-related gene expression in the liver (d) and Epi WAT (e). *n* = 6-8 per group.

Both diet-induced hepatosteatosis and adipogenesis would contribute to insulin resistance, which could eventually disrupt glucose metabolism and homeostasis. Here, we found that low-dose GA administration did not change plasma glucose levels during WTD feeding (Figure 4(a)). Glucose tolerance test performed at 6 weeks on the WTD feeding showed that low-dose GA administration did not significantly improve WTD-induced inhibition of glucose clearance (Figures 4(b) and 4(c)). Further, using real-time PCR analysis, we showed that low-dose GA administration did not significantly change the hepatic or adipose expression of insulin resistance-associated genes, such as Akt2, Irs1, and Irs2 (Figures 4(d) and 4(e)).

### 3.5. Low-Dose GA Administration Produces No Significant Benefit against Diet-Induced Atherogenesis

**Figure 5:**
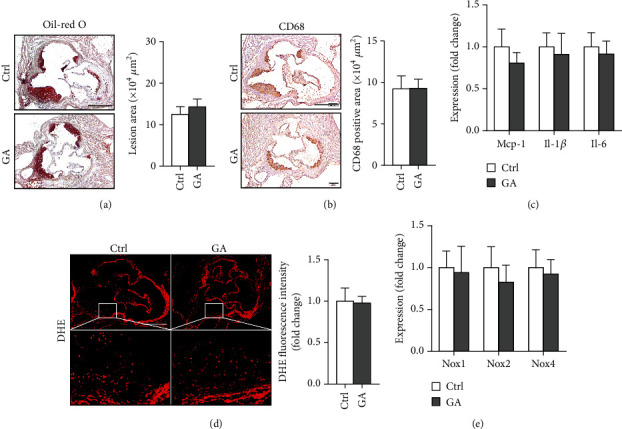
Effect of low-dose GA administration on diet-induced atherosclerosis in the Apoe KO mice. (a) Representative Oil-red O staining images of aortic root section and quantitation of aortic atherosclerotic plaque areas. (b) Representative CD68 immunochemical staining images of aortic root section and quantitation of CD68 positive macrophages infiltrated into the atherosclerotic plaques. (c) Quantitative real-time PCR analysis of macrophage inflammation-related gene expression in the aorta. (d) Representative DHE staining images of aortic root section and quantitation of DHE fluorescence intensity. The bar represents 500 *μ*m. (e) Quantitative real-time PCR analysis of Noxs gene expression in the aorta. *n* = 6-8 per group.

Finally, we showed here that low-dose GA administration did not reduce aortic atherosclerotic burdens, as shown by Oil-red O staining (Figure 5(a)). Using immunochemical staining with anti-CD68 antibody, we demonstrated that low-dose GA administration had no significant inhibitory effect on inflammatory CD68 positive macrophage infiltration into the plaque (Figure 5(b)). The anti-inflammatory potent of low-dose GA administration was further confirmed by real-time PCR, which showed no alteration of aortic expression of macrophage inflammatory cytokines, such as Mcp-1, Il-1*β*, and Il-6 (Figure 5(c)). Furthermore, low-dose GA administration did not significantly attenuate WTD-induced oxidative stress, shown by DHE staining (Figure 5(d)) and real-time PCR detecting the aortic gene expression of NADPH oxidase (Nox) subunits, including Nox1, Nox2, and Nox4 (Figure 5(e)).

## 4. Discussion

Plant-derived polyphenolic GA has been demonstrated for multiple pharmacological properties and therapeutic potent in cardiovascular diseases, such as diabetic cardiac remodeling and pressure overload-induced cardiac hypertrophy. In this study, we explored the *in vivo* effects of low-dose (20 mg/kg body weight daily) GA on maintaining metabolic homeostasis and preventing diet-induced atherosclerosis, using Apoe KO mice fed on high-fat WTD as disease models. Compared with wild-type mice, which are naturally resistant to ASCVD, Apoe mice could develop severe hypercholesteremia, insulin resistance, and atherosclerosis in 2-3 months of WTD feeding, therefore widely accepted as a small animal model for diet-induced metabolic disorders and atherosclerosis [13–15]. Unexpectedly, we did not observe that this dosage of GA administration could produce significant benefits against WTD-induced metabolic disorders, including hyperlipidemia, hepatosteatosis, adipogenesis, or insulin resistance; in addition, this dosage of GA administration also could not prevent WTD-induced atherosclerosis.

The potent of GA in maintaining metabolic homeostasis has been explored previously. In a mouse model of diet-induced nonalcoholic fatty liver disease, GA has been demonstrated to ameliorate diet-induced hypercholesterolemia, hepatosteatosis, obesity, and insulin resistance, possibly by correcting disturbances of several metabolic pathways involving lipid and glucose (glycolysis and gluconeogenesis) metabolism as well as amino acids, choline, and gut-microbiota-associated metabolism [16]. Notably, similar metabolic-protective effects of GA can be seen in another mouse model of diet-induced nonalcoholic fatty liver disease combined with streptozotocin-induced type II diabetes [17]. However, in the current study, we do not observe significant benefits of low-dose GA administration on diet-induced metabolic disorders, including hyperlipidemia (Figure 1), hepatosteatosis (Figure 2), adipogenesis (Figure 3), or insulin resistance (Figure 4), in the Apoe KO mouse models fed with high-fat WTD. Unlike regular wild-type mouse or rat models used in previous studies, Apoe KO mice develop spontaneous mild hypercholesterolemia (400-500 mg/dl) and atherosclerosis even on standard rodent chow diet [13]. High-fat WTD feeding induces a further increase of plasma cholesterol (800-1500 mg/dl) in Apoe KO mice, which is several times higher than those of wild-type mice and rats fed with the same type of diet [15]. In this severe hypercholesterolemia context, we hypothesize that the potent of GA might be insufficient to exert significant beneficial impact on systemic metabolism in the Apoe KO mice.

Atherosclerosis is a type of lipid-driven vascular injury that characterized by progressive inflammation and oxidative stress [18]. Phytochemicals with anti-inflammation and antioxidative properties are therefore potential therapeutic interventions for preventing and controlling atherosclerosis. As a well-known anti-inflammatory and antioxidative plant-derived polyphenolic acid, GA has already been demonstrated to provide multiple benefits in several cardiovascular disease conditions. For example, in a rat model of ischemia-reperfusion injury, GA is able to protect against cardiac oxidative stress and inflammation triggered by particulate matter containing PM10 [19]. Recently, in a mouse model of pressure overload-induced cardiac hypertrophy, GA administration also effectively attenuates cardiac inflammation and oxidative stress, therefore inhibiting the progression of myocardial fibrosis and heart failure [9]. In addition to cardiovascular disorders, the therapeutic potent of GA also extends to other types of diseases, such as cancers [20] and neurodegenerative diseases [21, 22]. Although GA possess broad-spectrum anti-inflammatory and antioxidative effects, whether GA could protect against atherosclerotic cardiovascular diseases is still unknown. In the current study, we demonstrate that low-dose GA administration does not reduce atherosclerotic plaque burden in the Apoe KO mice fed with the high-fat WTD, as shown by Oil-red O staining (Figure 5(a)). Furthermore, low-dose GA administration does not inhibit plaque inflammation, as shown by CD68 positive macrophage infiltration and inflammatory cytokine expressions (Figures 5(b) and 5(c)). Low-dose GA administration also does not attenuate plaque oxidative stress, as shown by DHE staining and Nox gene expressions (Figures 5(d) and 5(e)). Therefore, the anti-inflammatory and antioxidative potent of low-dose GA might be insufficient to exert significant beneficial impact on diet-induced atherosclerosis in the Apoe KO mice with severe systemic metabolic disorders.

In the current study, GA is administrated by daily oral gavage at a dosage of 20 mg/kg body weight, as a recent study has demonstrated that this dosage is effective to suppress cardiac hypertrophy and following heart failure induced by angiotensin ІІ or transverse aortic constriction [9]. In fact, the *in vivo* dosage of GA used in mouse study ranges at least from 2 mg/kg body weight to 100 mg/kg body weight [23]; therefore, the dosage we used in the current study is relatively low. In addition to dosage, disease and nutritional states that alter the normal gut function/microbiota or xenobiotic metabolizing systems might also affect the stability, absorption, and metabolism of the phytochemicals [24]. For examples, myocardial infarction is reported to hinder the absorption of GA [25] while diabetes accelerates the clearance of flavone glycoside baicalin as well as phenolic-like alkaloid jatrorrhizine in rats [26, 27]. Previous studies have well demonstrated that high-fat diet feeding increases intestinal permeability and change gut microbiota diversity and metabolome profile [28–30]. Whether the altered gut microenvironment caused by high-fat WTD feeding could alter the bioavailability and pharmacokinetics of GA that finally contributes to the ineffectiveness of low-dose GA to influence the progression of atherosclerotic cardiovascular diseases as seen in the Apoe KO mice therefore needs to be defined in the future.

In conclusion, we show here that low-dose polyphenolic GA produces no significant benefits against diet-induced metabolic disorders, including hyperlipidemia, hepatosteatosis, adipogenesis, or insulin resistance, as well as diet-induced atherosclerosis in the Apoe KO mice. Whether GA is beneficial for atherosclerotic cardiovascular diseases needs further exploration.

## Figures and Tables

**Table 1:** Primer sequences used in the study.

Gene	Forward (5′ toward 3′)	Reverse (5′ toward 3′)
Srebp1c	GACCACTCGCATTCCTTT	CCACAGACTCGGCACTCA
Dgat1	ATCTGAGGTGCCATCGTC	ATGCCATACTTGATAAGGTTCT
Dgat2	ATGTTCCCGAGGGAGACCAA	GAGGCTCCGTAGATGTGAGTG
Fasn	CTCCCGATTCATAATTGGGTCTG	TCGACCTTGTTTTACTAGGTGC
Acc1	CGCTGGCACATCAACTTCAC	AGGAACTCAGAAGCCCAAAGC
Scd1	GCGCTACTTCCGAGACTACTT	GGGCCTTATGCCAGGAAACT
Ppar*γ*	GTTAGCCATGTGGTAGGAGACA	CCCAGCCGTTAGTGAAGAGT
Pgc1*α*	TATGGAGTGACATAGAGTGTGCT	GTCGCTACACCACTTCAATCC
Ppar*α*	GGGCTTTCGGGATAGTTG	ATTGGGCTGTTGGCTGAT
Hsl	GGAGCCATCTTTGAGCCTTCA	GAACCAAACTGAGGAATGGATCT
Mttp	ATACAAGCTCACGTACTCCACT	TCTCTGTTGACCCGCATTTTC
Chrebp1	AGCATCGATCCGACACTCAC	TTGTTCAGCCGGATCTTGTC
Lxr	GCGACAGTTTTGGTAGAGGGAC	CGCTTTTGTGGACGAAGCTC
Npc1	CTGTGACCTGATCCCTACCC	CCTGTCTTCCCGGGCCATAA
Lrp1	CTCCCACCGCTATGTGATCC	CACAGCTGTTGGTGTCGTTG
Srb1	CGAAGTGGTCAACCCAAACGA	CCATGCGACTTGTCAGGCT
Abca1	AAAACCGCAGACATCCTTCAG	CATACCGAAACTCGTTCACCC
Acat1	CAGGAAGTAAGATGCCTGGAAC	TGCAGCAGTACCAAGTTTAGTG
Cebpa	GGGTCTATGCCACGATTC	GTGTCCCATGTTGGATTTG
Atgl	GATTTACGCACGATGACACAGT	ACCTGCAAAGACATTAGACAGC
Akt2	CAGATGGTCGCCAACAGT	TGCCGAGGAGTTTGAGATA
Irs1	GGATCGTCAATAGCGTAA	GCTTGGCACAATGTAGAA
Irs2	GGGGCGAACTCTATGGGTA	GCAGGCGTGGTTAGGGAAT
G6pase	AATCTCCTCTGGGTGGCA	GCTGTAGTAGTCGGTGTCC
Pepck	AGTCATCATCACCCAAGAGC	CCACCACATAGGGCGAGT
Glut4	ACGGATAGGGAGCAGAAA	AAGGGTGAGTGAGGCATT
Mcp-1	TAAAAACCTGGATCGGAACCAAA	GCATTAGCTTCAGATTTACGGGT
Il-1*β*	CTTCCCCAGGGCATGTTAAG	ACCCTGAGCGACCTGTCTTG
Il-6	TTCCATCCAGTTGCCTTCTTG	TTGGGAGTGGTATCCTCTGTGA
Nox1	CCCATCCAGTCTCCAAACATGAC	ACCAAAGCTACAGTGGCAATCAC
Nox2	CTTCTTGGGTCAGCACTGGC	GCAGCAAGATCAGCATGCAG
Nox4	CTTGGTGAATGCCCTCAACT	TTCTGGGATCCTCATTCTGG
Gapdh	TGATGACATCAAGAAGGTGGTGAAG	TCCTTGGAGGCCATGTAGGCCAT

## Data Availability

The data used to support the findings of this study are included within the article.

## Abbreviations

ASCVD: Atherosclerotic cardiovascular diseases
GA: Gallic acid
Apoe: Apolipoprotein E
KO: Knockout
WTD: Western-type diet
TC: Total cholesterol
TG: Triglycerides
HDL-C: High-density lipoprotein cholesterol
LDL-C: Low-density lipoprotein cholesterol
FPLC: Fast protein liquid chromatography
H&E: Hematoxylin and eosin
DHE: Dihydroethidium
WAT: White adipose tissue
suQ: Subcutaneous WAT
Epi: Epididymal WAT
Met: Mesenteric WAT
Ret: Retroperitoneal WAT
Nox: NADPH oxidase.

## References

[1] Mensah G. A., Roth G. A., Fuster V. (2019). The global burden of cardiovascular diseases and risk factors: 2020 and beyond. *Journal of the American College of Cardiology*.

[2] Lechner K., von Schacky C., McKenzie A. L. (2020). Lifestyle factors and high-risk atherosclerosis: pathways and mechanisms beyond traditional risk factors. *European Journal of Preventive Cardiology*.

[3] Khurana S., Venkataraman K., Hollingsworth A., Piche M., Tai T. C. (2013). Polyphenols: benefits to the cardiovascular system in health and in aging. *Nutrients*.

[4] Cory H., Passarelli S., Szeto J., Tamez M., Mattei J. (2018). The role of polyphenols in human health and food systems: a mini-review. *Frontiers in Nutrition*.

[5] Badhani B., Sharma N., Kakkar R. (2015). Gallic acid: a versatile antioxidant with promising therapeutic and industrial applications. *RSC Advances*.

[6] Patel S. S., Goyal R. K. (2011). Cardioprotective effects of gallic acid in diabetes-induced myocardial dysfunction in rats. *Pharmacognosy Research*.

[7] Ryu Y., Jin L., Kee H. J. (2016). Gallic acid prevents isoproterenol-induced cardiac hypertrophy and fibrosis through regulation of JNK2 signaling and Smad3 binding activity. *Scientific Reports*.

[8] Jin L., Sun S., Ryu Y. (2018). Gallic acid improves cardiac dysfunction and fibrosis in pressure overload- induced heart failure. *Scientific Reports*.

[9] Yan X., Zhang Y. L., Zhang L. (2019). Gallic acid suppresses cardiac hypertrophic remodeling and heart failure. *Molecular Nutrition & Food Research*.

[10] Liao J., Liu X., Gao M. (2018). Dyslipidemia, steatohepatitis and atherogenesis in lipodystrophic apoE deficient mice with Seipin deletion. *Gene*.

[11] Liu L., Liang C., Wang X. (2019). Surgical fat removal exacerbates metabolic disorders but not atherogenesis in LDLR^−/−^ mice fed on high-fat diet. *Scientific Reports*.

[12] Zhang Y., An X., Lin Q., Bai J., Wang F., Liao J. (2018). Splenectomy had no significant impact on lipid metabolism and atherogenesis in Apoe deficient mice fed on a severe atherogenic diet. *Cardiovascular Pathology*.

[13] Plump A. S., Smith J. D., Hayek T. (1992). Severe hypercholesterolemia and atherosclerosis in apolipoprotein E-deficient mice created by homologous recombination in ES cells. *Cell*.

[14] Liao J., Huang W., Liu G. (2017). Animal models of coronary heart disease. *Journal of Biomedical Research*.

[15] Veseli B. E., Perrotta P., De Meyer G. R. (2017). Animal models of atherosclerosis. *European Journal of Pharmacology*.

[16] Chao J., Huo T. I., Cheng H. Y. (2014). Gallic acid ameliorated impaired glucose and lipid homeostasis in high fat diet-induced NAFLD mice. *PLoS One*.

[17] Chao J., Cheng H. Y., Chang M. L. (2020). Gallic acid ameliorated impaired lipid homeostasis in a mouse model of high-fat diet-and streptozotocin-induced NAFLD and diabetes through improvement of beta-oxidation and ketogenesis. *Frontiers in Pharmacology*.

[18] Ross R. (1999). Atherosclerosis--an inflammatory disease. *The New England Journal of Medicine*.

[19] Radan M., Dianat M., Badavi M., Mard S. A., Bayati V., Goudarzi G. (2019). Gallic acid protects particulate matter (PM10) triggers cardiac oxidative stress and inflammation causing heart adverse events in rats. *Environmental Science and Pollution Research International*.

[20] Ashrafizadeh M., Zarrabi A., Mirzaei S. (2021). Gallic acid for cancer therapy: molecular mechanisms and boosting efficacy by nanoscopical delivery. *Food and Chemical Toxicology*.

[21] Schaffer S., Asseburg H., Kuntz S., Muller W. E., Eckert G. P. (2012). Effects of polyphenols on brain ageing and Alzheimer’s disease: focus on mitochondria. *Molecular Neurobiology*.

[22] Devi S. A., Chamoli A. (2020). Polyphenols as an effective therapeutic intervention against cognitive decline during normal and pathological brain aging. *Advances in Experimental Medicine and Biology*.

[23] Dludla P. V., Nkambule B. B., Jack B. (2018). Inflammation and oxidative stress in an obese state and the protective effects of gallic acid. *Nutrients*.

[24] Redan B. W., Buhman K. K., Novotny J. A., Ferruzzi M. G. (2016). Altered transport and metabolism of phenolic compounds in obesity and diabetes: implications for functional food development and assessment. *Advances in Nutrition*.

[25] Yu Z., Song F., Jin Y.-C. (2018). Comparative pharmacokinetics of gallic acid after oral administration of gallic acid monohydrate in normal and isoproterenol-induced myocardial infarcted rats. *Frontiers in Pharmacology*.

[26] Liu L., Deng Y.-X., Liang Y. (2010). Increased oral AUC of baicalin in streptozotocin-induced diabetic rats due to the increased activity of intestinal *β*-glucuronidase. *Planta Medica*.

[27] Yu S., Yu Y., Liu L. (2010). Increased plasma exposures of five protoberberine alkaloids from Coptidis Rhizoma in streptozotocin-induced diabetic rats: is P-GP involved?. *Planta Medica*.

[28] Bibbò S., Ianiro G., Giorgio V. (2016). The role of diet on gut microbiota composition. *European Review for Medical and Pharmacological Sciences*.

[29] De Angelis M., Garruti G., Minervini F., Bonfrate L., Portincasa P., Gobbetti M. (2019). The food-gut human axis: the effects of diet on gut microbiota and metabolome. *Current Medicinal Chemistry*.

[30] Rohr M. W., Narasimhulu C. A., Rudeski-Rohr T. A., Parthasarathy S. (2020). Negative effects of a high-fat diet on intestinal permeability: a review. *Advances in Nutrition*.

